# Supplementary material for: Social prescribing for children and youth: A scoping review protocol
Source: PLoS One. 2024 Mar 8;19(3):e0297535. doi: 10.1371/journal.pone.0297535 (PMC10923428; doi:10.1371/journal.pone.0297535)
Supplement: S1 File — (PDF) [file pone.0297535.s001.pdf]

Appendix I: Recommended items to address in a scoping review protocol*

Section and topic	Item No	Checklist item	Page#
ADMINISTRATIVE INFORMATION			
Title:			
Identification	1a	Identify the report as a protocol of a scoping review	1
Update	1b	If the protocol is for an update of a previous scoping review, identify as such	N/A
Registration	2	If registered, provide the name of the registry (such as JBI) and registration number	7
Authors:			
Contact	3a	Provide name, institutional affiliation, email address of all protocol authors; provide physical mailing address of corresponding author	Submission System
Contributions	3b	Describe contributions of protocol authors and identify the guarantor of the review	Submission System
Amendments	4	If the protocol represents an amendment of a previously completed or published protocol, identify as such and list changes; otherwise, state plan for documenting important protocol amendments	N/A
Support:			
Sources	5a	Indicate sources of financial or other support for the review	N/A
Sponsor	5b	Provide name for the review funder and/or sponsor	N/A
Role of sponsor or funder	5c	Describe roles of funder(s), sponsor(s), and/or institution(s), if any, in developing the protocol	N/A
INTRODUCTION			
Rationale	6	Describe the rationale for the review in the context of what is already known (Note: Consider providing a rationale for the choice of conducting a scoping review as compared to other evidence synthesis approaches)	3-5
Objectives	7	Provide an explicit statement of the question(s) the review will address with reference to the inclusion/ exclusion criteria	5
METHODS			
Eligibility criteria	8	Specify the study characteristics (such as PICO, study design, setting, timeframe) and report characteristics (such as years considered, language, publication status) to be used as criteria for eligibility for the review	5-6
Information sources	9	Describe all intended information sources (such as electronic databases, contact with study authors, trial registers or other gray literature sources) with planned dates of coverage	9
Search strategy	10	Present draft of search strategy to be used for at least one electronic database, including planned limits, such that it could be repeated	7-8
Study records:			
Data management	11a	Describe the mechanism(s) that will be used to manage records and data throughout the review	9-10
Selection process	11b	State the process that will be used for selecting studies (such as two independent reviewers) through each phase of the review (that is, screening, eligibility and inclusion)	9-10
Data collection process	11c	Describe planned method of extracting data from reports (such as piloting forms, done independently, in duplicate), any processes for obtaining and confirming data from investigators	10
Data items	12	List and define all variables for which data will be sought (such as PICO items, funding sources), any preplanned data assumptions and simplifications (Note: Scoping reviews may not use PICO and instead may use JBI's Population, Concept, and Context [PCC] or another approach to reporting eligibility criteria)	10
Outcomes and prioritization	13	List and define all outcomes for which data will be sought, including prioritization of main and additional outcomes, with rationale (Note: Scoping reviews may not extract outcome data, so this can refer to whichever data items are extracted)	10

(*Continued*)			
**Section and topic**	**Item No**	**Checklist item**	**Page#**
Risk of bias in individual studies	14	If this is to occur, describe anticipated methods for assessing risk of bias of individual studies, including whether this will be done at the outcome or study level, or both; state how this information will be used in data synthesis (Note: Scoping reviews typically do not include risk of bias assessment, but this information should be described if it will occur)	N/A
Data synthesis	15a	Describe criteria under which study data will be presented (Note: Scoping reviews do not typically include quantitative synthesis of study data, but should still describe in advance how extracted data are anticipated to be presented in the resulting review)	11
	15b	Describe the planned approach to how extracted data will be presented (such as figures, tables, evidence gaps maps)	11
	15c	Describe any proposed additional analyses (such as thematic analyses) (Note: The JBI methodological guidance does not recommend undertaking thematic analysis as this synthesis of data should ideally occur following methodological appraisal of the included sources)	N/A
	15d	If quantitative synthesis is not appropriate, describe the type of summary planned	N/A
Meta-bias(es)	16	Specify any planned assessment of meta-bias(es) (such as publication bias across studies, selective reporting within studies) (Note: Scoping reviews typically do not include assessment of meta-bias[es], but this information should be described if it will occur)	N/A
Confidence in cumulative evidence	17	Describe how the strength of the body of evidence will be assessed (such as GRADE)	N/A

GRADE, Grading of Recommendations, Assessment, Development and Evaluation; PICO, participants, intervention, comparator, outcomes; PRISMA, Preferred Reporting Items for Systematic Reviews and Meta-Analyses.

From: Shamseer L, Moher D, Clarke M, Ghersi D, Liberati A, Petticrew M, *et al.*; PRISMA-P Group. Preferred reporting items for systematic review and meta-analysis protocols (PRISMA-P) 2015: elaboration and explanation. BMJ. 2015;349:g7647. CC BY 4.0.

*—This checklist is based on the PRISMA for systematic review protocols (PRISMA-P) checklist.[i] It is strongly recommended that this checklist be read in conjunction with the best practice guidance and reporting items for the development of scoping review protocols for important clarification on the items, [ii] and the JBI updated methodological guidance for the conduct of scoping reviews.[iii] Amendments to a scoping review protocol should be tracked and dated.

[i]Moher D, Shamseer L, Clarke M, Ghersi D, Liberati A, Petticrew M, *et al.* Preferred Reporting Items for Systematic Review and Meta-Analysis Protocols (PRISMA-P) 2015 statement. Syst Rev. 2015;4(1):1.

[ii]Peters MDJ, Godfrey C, McInerney P, Khalil H, Larsen P, Marnie C, *et al.* Best practice guidance and reporting items for the development of scoping review protocols. JBI Evid Synth. 2021. [Epub ahead of print]

[iii]Peters MDJ, Marnie C, Tricco AC, Pollock D, Munn Z, Alexander L, *et al.* Updated methodological guidance for the conduct of scoping reviews. JBI Evid Synth. 2020;18 (10):2119-26.
