# Supplementary material for: Social prescribing for children and youth: A scoping review protocol
Source: PLoS One. 2024 Mar 8;19(3):e0297535. doi: 10.1371/journal.pone.0297535 (PMC10923428; doi:10.1371/journal.pone.0297535)
Supplement: Attachment — Submitted filename: Response to Reviewers.docx [file pone.0297535.s002.docx]

**Social Prescribing for Children and Youth: A Scoping Review Protocol – Response to Reviewers**

Thank you for providing us with valuable feedback which has helped us to enhance our manuscript. We have made the necessary changes and have also made a few minor updates to the protocol.

| **Academic Editor Comments** | **Response** |
| --- | --- |
| When submitting your revision, we need you to address these additional requirements.  1. Please ensure that your manuscript meets PLOS ONE's style requirements, including those for file naming. The PLOS ONE style templates can be found at  <https://journals.plos.org/plosone/s/file?id=wjVg/PLOSOne_formatting_sample_main_body.pdf> and  <https://journals.plos.org/plosone/s/file?id=ba62/PLOSOne_formatting_sample_title_authors_affiliations.pdf>  2. Please include captions for your Supporting Information files at the end of your manuscript, and update any in-text citations to match accordingly. Please see our Supporting Information guidelines for more information: <http://journals.plos.org/plosone/s/supporting-information>.  3. We note that this manuscript is a systematic review or meta-analysis; our author guidelines therefore require that you use PRISMA guidance to help improve reporting quality of this type of study. Please upload copies of the completed PRISMA checklist as Supporting Information with a file name “PRISMA checklist”. | Thank you for outlining these requirements.  We have updated the manuscript to ensure that it meets the style requirements.  We have updated the manuscript to incorporate our Supporting Information file.  Since this is a scoping review protocol, we did not include a PRISMA checklist because that checklist is specifically for systematic review and meta-analysis protocols. Instead, we included a checklist that was developed by JBI for scoping review protocols. This checklist has been included as Supporting Information with a file name “S1_File”. |
| The reviewers generally concur on the valid rationale behind the protocol's topic. One of the reviewers indicate that additional justification of the publication of the protocol is required. However, no modifications are required to address this concern.  The editorial staff has brought to my attention that there is a related Study Protocol currently under consideration at PLOS ONE titled "Social Prescribing and Students: A Scoping Review Protocol," which delves into the examination of evidence regarding social prescribing and students. It is essential to emphasize that the journal strongly discourages the unnecessary fragmentation of related work into separate manuscripts. PLOS ONE's policy mandates that each submission be a self-contained unit, without reliance on work that has not yet been accepted for publication. If authors choose to submit related manuscripts to PLOS ONE, they are strongly encouraged to assess the potential for consolidation into a single comprehensive manuscript. Alternatively, authors should provide compelling reasons for separate publication. In view of this, a major revision decision has been given to your manuscript at this time. | We value the feedback from the reviewers and have responded to their comments.  We want to alleviate your concerns about the two protocols. These are completely separate pieces of work. Please see our detailed explanation below.  The first author will be conducting the scoping review on social prescribing for children and youth as part of their doctoral studies. In this role, the first author is leading this review with support from their supervisory committee and librarian. As highlighted in the manuscript, there is a need for a review of the evidence on social prescribing for children and youth. As such, this scoping review will examine the evidence that exists on social prescribing programs that target children and youth.  The first author is also one of the co-leads of a national social prescribing student group. In this role, the first author is collaborating with other members of the student group to conduct the scoping review on social prescribing and students. This is a completely separate review being conducted for a different purpose on a different topic with different people. As highlighted in the manuscript, there is a need for a review of the evidence on social prescribing and students. As such, this scoping review is examining the evidence that exists on social prescribing **and** students, such as social prescribing programs **for** students, social prescribing programs **by** students, social prescribing programs **with** students, and efforts to educate students about social prescribing. We also note that age is not considered in this review as eligibility is determined solely based on the use of the term ‘student’. This means that the population of interest is not the same as the review on social prescribing for children and youth, which is limited by age ($\leq$25 years).  There are some similarities between the protocols, but this is to be expected. Both of these manuscripts have been prepared in accordance with JBI methodology, which has very specific requirements around what needs to be included in scoping review protocols. Additionally, both of these manuscripts focus on social prescribing, hence the similarity in methods. Overall, the similarities between the manuscripts simply demonstrate that we are following JBI methodology and approaching the topic of social prescribing in a consistent way.  In sum, we want to emphasize that these are completely separate pieces of work, with both of them warranting completion given the lack of evidence reviews in these areas. |

| **Reviewer 1 Comments** | **Response** |
| --- | --- |
| Social prescribing has been implemented as a novel intervention to address non-clinical social needs with promising results. Children and adolescents do have non clinical social needs and it is important that literature on this intervention in this population is explored. A scoping review is appropriate in this case. The objectives and methods have been well explained. | Thank you kindly. |

| **Reviewer 2 Comments** | **Response** |
| --- | --- |
| The protocol does have a valid rationale, however, it is not clear why there is a need for the authors to have their protocol published before they start with their actual reviews. I do not see this as a topical issue that can be at risk if not published from the protocol.  The methodology is not clearly outlined and described, and this makes it difficult to fully understand the protocol. | Thank you for your feedback. As mentioned in the manuscript, we feel it is important to publish this protocol to promote transparency. Going through the publishing process also offers a valuable learning opportunity for the first author, who is doing this work as part of their doctoral studies. We note that JBI emphasizes that protocols should be published, registered, or made publicly available. Please see relevant excerpts from JBI below.  *“The protocol is a valuable manuscript to publish or make publicly available because it informs the research community of the intentions and focus of the review authors prior to the conduct of the review itself.”*  Peters, M. D. J., Godfrey, C., McInerney, P., Khalil, H., Larsen, P., Marnie, C., Pollock, D., Tricco, A. C., & Munn, Z. (2022). Best practice guidance and reporting items for the development of scoping review protocols. JBI evidence synthesis, 20(4), 953–968. https://doi.org/10.11124/JBIES-21-00242  *“Authors conducting a scoping review should consider publishing, registering, or making their protocol available via platforms...”*  Peters, M. D. J., Marnie, C., Tricco, A. C., Pollock, D., Munn, Z., Alexander, L., McInerney, P., Godfrey, C. M., & Khalil, H. (2020). Updated methodological guidance for the conduct of scoping reviews. JBI evidence synthesis, 18(10), 2119–2126. https://doi.org/10.11124/JBIES-20-00167  The creation of this protocol was informed by best practice guidance for scoping review protocols from the JBI Scoping Review Methodology Group, ensuring that all of the key components of scoping review methodology have been considered. We have added some additional details to the manuscript and have referenced papers that explain scoping review methodology. |

| **Reviewer 3 Comments** | **Response** |
| --- | --- |
| This is a clear and well written protocol for this scoping review. The authors rightly point out that less attention has been given to this age group and therefore this review is warranted. Furthermore, it will be valuable to apply the operational constructs of their recently published definition to the included studies.  There was a brief description of how data will be presented, but I couldn't see where data underlying the findings will be made available once the study is complete.  I have no further comments and wish the authors good luck with their work. | Thank you kindly.  We have updated the manuscript to explain where data underlying the findings will be made available once the study is complete.  “This review will be published in a peer-reviewed journal. All data relevant to the study will be included in the article or uploaded as supplementary information.” (lines 162-164) |
